# Reliability and usefulness of spirometry performed during admission for COPD exacerbation

**DOI:** 10.1371/journal.pone.0194983

**Published:** 2018-03-26

**Authors:** Alberto Fernández-Villar, Cristina Represas-Represas, Cecilia Mouronte-Roibás, Cristina Ramos-Hernández, Ana Priegue-Carrera, Sara Fernández-García, José Luis López-Campos

**Affiliations:** 1 Pulmonary Department, Álvaro Cunqueiro Hospital, EOXI, Vigo, Spain; 2 NeumoVigoI+i Research Group, Instituto de Investigación Sanitaria Galicia Sur (IISGS), Vigo, Spain; 3 Medical-Surgical Unit of Respiratory Diseases, Sevilla Biomedical Research Institute (IBiS), Virgen del Rocío University Hospital, Sevilla University, Spain; 4 CIBER of Respiratory Diseases (CIBERES), Health Institute Carlos III, Madrid, Spain; National and Kapodistrian University of Athens, GREECE

## Abstract

**Objectives:**

Although not currently recommended, spirometry during hospitalization due to exacerbation of chronic obstructive pulmonary disease (COPD) is an opportunity to enhance the diagnosis of this disease. The aim of the present study was to assess the usefulness and reliability of spirometry before hospital discharge, comparing it to measurements obtained during clinical stability.

**Methods:**

This prospective longitudinal observational study compares spirometry results before and 8 weeks after discharge in consecutive patients admitted for COPD exacerbation. Concordance between results was assessed by the Kappa index, intraclass correlation coefficient, and Bland-Altman graphs.

**Results:**

From an initial population of 179 COPD patients, 100 completed the study (mean age 67.8 years, 83% men, 35% active smokers, FEV_1_ at clinical stability 40.3%). Forty-nine patients could not complete the study because they did not reach clinical stability. In three patients with obstructive spirometry during admission, the results were normal at follow-up. In the remaining patients, the COPD diagnosis was confirmed at stability with acceptable concordance. In 27 cases, spirometry improved more than 200 mL.No variables were found to be associated with this improvement or to explain it.

**Conclusions:**

This study provides information on the role of spirometry prior to hospital discharge in patients admitted for COPD exacerbation, demonstrating that it is a valid and reproducible method, representing an opportunity toimprove COPD diagnosis.

## Introduction

Chronic obstructive pulmonary disease (COPD) is a chronic disease with high prevalence and mortality, leading to significant health costs [1–3]. The diagnosis of COPD is based on inhaled exposure to different toxins, mainly tobacco, the presence of respiratory symptoms, and the demonstration of chronic airflow obstruction [1,2]. Although COPD is remarkably underdiagnosed, imprecision in this diagnosis has been described [4,5].

Spirometry is essential to assessing airflow obstruction and COPD diagnosis. The results of this measurement play a fundamental role in scales and classifications of the disease, making it essential to patient characterization. However, spirometry is widely underused [6]. Consequently, it is not uncommon for a patient to be diagnosed with COPD only on the basis of clinical suspicion, without proper confirmation based on the demonstration of airflow obstruction with spirometry [7–9]. Consistently, spirometry confirms COPD diagnosis in 20–70% of patients [9–12].

Inadequate diagnosis of COPD is a considerable health problem, as it can lead to inadequate treatment prescriptions, with health costs and risks for patients, leading to delays in diagnosing and treating the true cause of the symptoms [5,9,12]. In addition, the lack of spirometric assessment increases the probability of readmission [13]. Therefore, hospital admission isa pertinent time to review diagnostic adequacy, confirming it when previous spirometry data are avalaible or actualizing lung function tests [9,14,15]. Spirometry before discharge may confirm the diagnosis, but current guidelines and recommendations do not include it within the protocols for managing COPD exacerbation; they advise postponing spirometry until stability is achieved [1,2] as usually done in general practice [10]. However, this is not always possible because a significant number of patients relapse or are readmitted in the weeks following hospitalization due to COPD exacerbation, are lost in the follow-up, or followed without spirometry [10,12,16].

Currently, evidence on the validity and reliability of spirometry performed during admission is scarce due to the heterogeneity and limitations of available studies [9,10,14,17,18]. Accordingly, we do not know whether differences between spirometry performed during or after hospitalization lead to changes in the symptoms or impact of COPD, or predict subsequent events in the course of the disease [14,17,18].

This study aims to provide evidence on these aspects after evaluating the reliability of spirometric parameters at hospital discharge and comparing them to those obtained in a stable phase. In addition, this study assesses whether any differences between the results imply significant changes in symptoms, alter the impact of COPD, or help predict subsequent events in the course of the disease.

## Methods

### Design and patient recruitment

This longitudinal prospective study was performed in a Spanish universitary hospital. The results of spirometry 24–48 hours before discharge and 8 weeks after discharge were compared. Between January 2014 and June 2016, consecutive patients admitted to our pneumology ward due to COPD exacerbation were included until the defined sample size was reached according to Global Initiative for Chronic Obstructive Lung Disease (GOLD) guidelines [1]. Patients with any psychic limitation that prevented them from performing the correct spirometric maneuvers, who presented with a contraindication for this test and those that were shown by spirometry to have no airflow obstruction before discharge,orwho presented with a new exacerbation in the first 8 weeks after discharge were excluded.

### Study protocol

Admission variables were collected 24–48 hours prior to discharge using a standardized questionnaire administered by a nurse specialized in research projects.

Variables included age, sex, education level (primary, secondary, or university), place of residence, personal history (current or former tobacco exposure, pack-years, previous asthma diagnosis, previous COPD diagnosis, and number of exacerbations and hospitalizations in the previous 12 months), clinical situation before discharge (Charlson Comorbidity Index, dyspnea according to the modified British Medical Research Council [mMRC] scale [19], and COPD Assessment Test [CAT] [20]), and data from discharge (pharmacological treatment after discharge and hospitalization duration in days).

Spirometry was performed after a bronchodilator test (30 min after administering nebulized 2.5 mg salbutamol and 0.5 mg ipratropium bromide) with a Datospir 120 spirometer (Sibelmed, Barcelona) in which we included the body mass index (BMI), forcedvital capacity (FVC), forced expiratory volume in the first second (FEV_1_), and the FEV_1_/FVC ratio. FVC and FEV_1_ values were collected as absolute and relative, as a percentage of the predicted value following Spanish reference values [21] and recommendations [22]. Relative FEV_1_ values were classified according to GOLD cut-offs [1] as mild (≥80%), moderate (50–79%), severe (30–49%), and very severe (<30%). Finally, blood count was obtained in order to determine the number of eosinophils.

Patients were reviewed at the outpatient clinic 8 (±1) weeks after discharge. The CAT and mMRC questionnaires, as well as a new spirometry test, were performed by the same nurse with the same spirometer. All spirometries (both prior to discharge and at follow-up) were performed during the mornings from 09:00 to 13:00 h. After this visit, monthly passive follow-up was carried out for a minimum of 6 months by reviewing electronic medical recordsin order to assess readmissions due to COPD exacerbation and the number of emergency room visits.

For the purpose of this study, an increase in FEV_1_ ≥ 200 mL was considered because it is usually significant and may be clinically important according to ERS/ATS recommendations [23].

### Statistical analysis

A descriptive analysis was performed for all included variables. Results were expressed as percentages and absolute frequencies for the qualitative variables and as mean and standard deviation for the numeric data. Data were analyzed using the statistical package IBM SPSS Statistics 21.0 (IBM Corporation, Armonk, NY). The numbers of readmissions and emergency room visitswererecorded absolutely and adjusted by the number of follow-up months for each patient for the comparative analysis in order toobtainan annualized value.

The student T test was used to comparequantitative variables between discharge and evaluation at 8 weeks. The other cross-sectional comparisons between numerical variables were performed using T tests for independent data after evaluatingvarianceequalitywith the Levene test. Chi-square or McNemar tests were used for qualitative variables in longitudinal or transversal studies, respectively.

Pearson correlation tests (r) estimating the determination coefficient (R^2^) were performed in order to study the association between both spirometry measurements. Their concordance at determining the existence and severity of airflow obstruction was evaluated using Cohen's Kappa index. The intraclass correlation coefficient was used with its 95% confidence interval (CI), as well as Bland Altman graphs, to assess the concordance of FVC and FEV_1_ measurements at discharge and follow-up.

Parameters obtained from a pilot study with the first 24 patients (data not published) were used to estimate the necessary sample size to find differences between spirometric values. An estimated 98 patients had to be recruited to achieve 80% power with a significance level of 5%.

### Ethics

This study was approved by the Galician Committee of Clinical Trials and Research (file number 2014/608). All patients provided written informed consent.

## Results

### Patient characteristics

**Fig 1:**
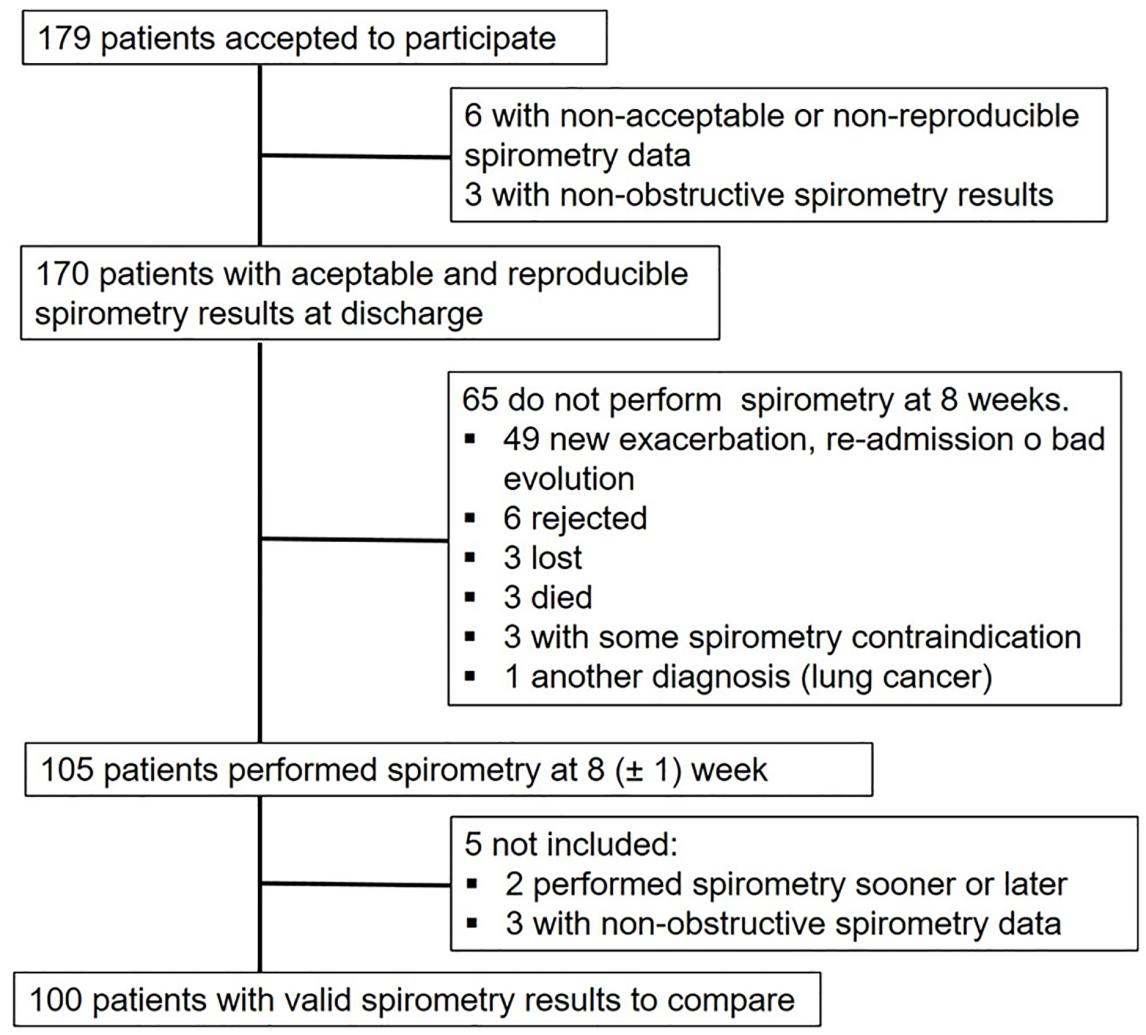
Patient flowchart.

The patient flowchart is given in Fig 1. A total of 179 consecutive patients were included after being admitted for COPD exacerbation. Valid maneuvers could be obtained in 170 (95%) cases. At 8 weeks, 3 (2.8%) patients were excluded after spirometry detected no obstruction. Another two patients were excluded because spirometry was performed at 4 and 12 weeks instead of 8 weeks by mistake.

The main characteristics of the 100 patients with an obstructive spirometry result who could be assessed at 8 weeks are described in Table 1. Three patients with an FEV_1_/FVC ratio of 65–69% in pre-discharge spirometry had no obstruction after 8 weeks. Of the 100 patients, 80 had COPD diagnosed based on obstructive spirometry and 3 on the basis of clinical criteria, whereas 17 had no previous diagnosis of any pulmonary obstructive disease.

**Table 1:** Demographic and clinical characteristics.

Variable	Global (n = 100)	FEV_1_ increased ≥ 200 mL at 8 weeks (n = 27)	FEV_1_ not increased ≥ 200 mL at 8 weeks (n = 73)	P-value
**Filiation**				
**Age, years**	67.8 (8.6)	69.1 (8.8)	67.3 (8.6)	0.36
**Sex (male), %**	83	81.5	83.6	0.77
**Rural residence, %**	35	34.6	36.6	0.85
**Primary studies, %**	79	81.5	79.2	0.83
**Background**				
**Current smoker, %**	35	48.1	30.6	0.20
**Tobacco exposure, pack-years**	52.4 (4.2)	54.1 (23.6)	51.9 (25.6)	0.70
**Confirmed diagnosis of COPD before admission, %**	80	74.1	83.6	0.17
**Asthma diagnosis before admission, %**	11.4	11.5	11.6	0.99
**Positive bronchodilator test before admission, %**	59.6	54.5	61.1	0.49
**Moderate and severe exacerbations in the last year**	2.7 (1.8)	2.7 (1.7)	2.7 (1.8)	0.83
**≥ 2 moderate and severe exacerbations in the last year**,%	66	77.3	63.6	0.16
**Situation prior to discharge**				
**Dyspnea, mMRC**	1.8 (0.9)	1.7 (0.8)	1.9 (0.9)	0.37
**CAT questionnaire**	15.3 (7.5)	14.6 (7.1)	15.8 (7.5)	0.45
**Charlson Comorbidity Index**	2.4 (1.5)	2.7 (1.6)	2.3 (1.4)	0.17
**Body mass index,kg/m**^**2**^	26.7 (5.5)	27.5 (6.1)	26.3 (5.3)	0.35
**FEV**_**1**_ **at discharge, L**	1.08 (0.4)	0.9 (0.34)	1.08 (0.4)	0.11
**FEV**_**1**_ **at discharge, %**	37.5 (14.8)	33.9 (12.2)	38.9 (15.6)	0.13
**FVC at discharge, L**	2.3 (0.6)	2.3 (0.6)	2.4 (0.6)	0.57
**FVC at discharge, %**	61.5 (15.6)	59.2 (15.9)	62.3 (15.5)	0.31
**Blood eosinophils,cells/μL**	90.1 (128.3)	118.9 (193.4)	79.2 (92.7)	0.30
**Blood eosinophils, %**	0.75 (0.75)	0.8 (0.8)	0.7 (0.7)	0.30
**IgE,UI/mL**	436.6 (914)	530.5 (1135.9)	389.6 (794.6)	0.58
**GOLD functional classification at discharge, %**				
**GOLD 2**	20	14.8	21.9	0.55
**GOLD 3**	37	33.3	38.4	
**GOLD 4**	43	51.9	39.7	
**GOLD 2017 classification at discharge, %**				
**GOLD C**	23	22.9	26.4	0.78
**GOLD D**	77	73.1	77.7	
**Discharge variables**				
**Length of hospital stay, days**	7.7 (4.2)	8.0 (4.3)	7.6 (4.5)	0.71
**Hospital stay until spirometry, days**	6.8 (4.8)	6.5 (3.5)	7.03 (5.3)	0.67
**Treatment at discharge, %**				
**LABA at discharge**	93	92.6	93.2	0.98
**LAMA at discharge**	88	85.2	88.8	0.73
**IC at discharge**	81	77.8	82.2	0.77
**Roflumilast at discharge**	10	3.7	12.3	0.28
**Teophilines at discharge**	14	11.5	15.1	0.75
**Home oxygen therapy**	39	33.3	41.1	0.64
**Non-invasive ventilation**	8	3.7	9.6	0.24

Data are expressed as mean (standard deviation) or absolute frequencies (relative).

LABA:long actingβ_2_agonist; LAMA: long acting muscarinic antagonist; IC: inhaled corticosteroid; GOLD: Global Initiative for Obstructive Lung Disease; CAT: COPD Assessment Test; mMRC: Modified British Medical Research Council dyspnea scale

### Spirometric changes

The mean values for spirometric parameters after bronchodilator administration at discharge and 8 weeks are givenin Table 2. These results show significant improvements in all parameters.

**Table 2:** Spirometric values before and 8 weeks after discharge.

Spirometric value	Prior to discharge	8 weeks after discharge	P-value
**FEV**_**1**_, **L**	1.04 (0.4)	1.12 (0.4)	0.005
**FEV**_**1**_, **%**	37.5 (14.8)	40.3 (16.5)	0.003
**FVC, L**	2.30 (0.6)	2.60 (0.7)	<0.001
**FVC**, **%**	61.5 (15.6)	66.8 (16.2)	<0.001
**FEV**_**1**_/**FVC**, **%**	43.6 (12.6)	43.5 (12.9)	0.949

Data are expressed as mean (standard deviation)

FEV_1_: forced expiratory volume in the first second; FVC: forced vital capacity.

The mean FEV1 improvement (SD) at 8 weeks of patients with a previous spirometric diagnosis was 0.048 (0.22) L versus 0.218 (0.41) L in those who did not have an obstructive spirometry before admission (p = 0.09).

**Fig 2:**
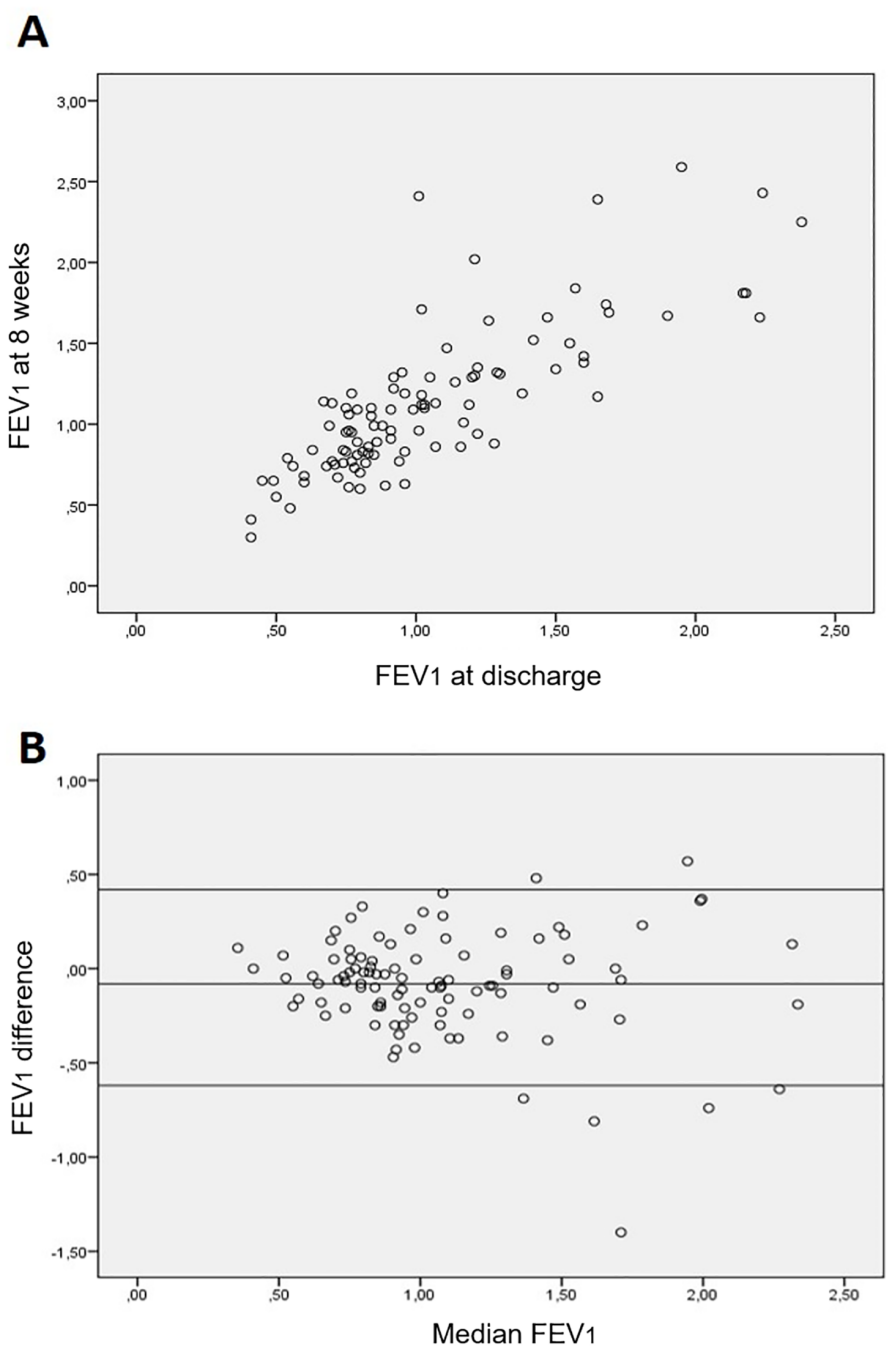
Correlation between FEV_1_ at discharge and 8 weeks. A. Linear correlation. B. Bland-Altman graph.

The correlation between FEV_1_ at discharge and 8 weeks (Fig 2A) was 0.804 (p<0.001), and R^2^ was 0.647 (p<0.01). The mean intraclass correlation coefficient was 0.890 (95% CI 0.837–0.926). The Bland-Altman graph is provided in Fig 2B, showing an acceptable concordance, grouping fairly symmetrically between established limits.

### Factors related to a ≥ 200 mL improvement in FEV_1_ at 8 weeks

FEV_1_ at 8 weeks increased ≥200 mL in 27% of patients. Variations in FEV_1_ according to GOLD classification at discharge and 8 weeks after are shown in Table 3. The number of patients with GOLD 2, GOLD 3, and GOLD 4 obstruction before discharge was 20%, 37%, and 43%, respectively, and 25%, 46%, and 29% after 8 weeks. The Kappa index was 0.588 (p<0.001). Based on the GOLD functional classification at discharge, the percentage of patients with an increase ≥ 200 mLin FEV_1_ was 20% for GOLD 2, 24.3% for GOLD 3, and 32.6% for GOLD 4. Table 1 shows the variables collected according to whether there was an increase in FEV_1_ levels 8 weeks after discharge; none of them were related to the analyzed improvement in spirometry results.

**Table 3:** Change in the GOLD functional classification according to spirometry performed at hospital discharge and 8 weeks.

Pre-discharge	At 8 weeks				
	FEV_1_ change,L	(%)	GOLD 2	GOLD 3	GOLD 4
**GOLD 2** **(n = 20)**	0.029 (0.32)	0.51 (10.5)	19 (95)	1 (5)	0 (0)
**GOLD 3** **(n = 37)**	0.077 (0.35)	2.67 (11.0)	6 (16.2)	28 (75.7)	3 (8.1)
**GOLD 4** **(n = 43)**	0.107 (0.16)	3.9 (5.9)	0 (0)	17 (39.5)	26 (60.5)
**Total** **(n = 100)**	0.808 (0.27)	2.77 (9.1)	25 (25)	46 (46)	29 (29)

Data are expressed as mean (standard deviation) or absolute frequencies (relative) according to the nature of the variables. Percentages refer to the number of patients per row.

FEV_1_: forced expiratory volume in the first second

### Clinical changes and their relation to FEV_1_

Patients with important increases in FEV_1_ had significant reductions in CAT scores compared to those without functional improvement [-4.07 (5.5) vs. -1.04 (5.3); p = 0.03]. However, there were no significant differences in the mMRC score [-0.05 (0.5) vs. -0.22 (0.6); p = 0.20]. The percentage of GOLD C and D patients according to the 2017 classification was 23% and 77% at discharge, and 24% and 76% at 8 weeks, respectively (p = 0.9). Ten patients classified as GOLD D at discharge were changed to GOLD C at 8 weeks, and 10 classified as GOLD D werechanged to GOLD C. The Kappa index was 0.415 (p<0.001).

### Follow-up

Patients were followed for 20 (9.5) months(range 6–38 months). The total number of readmissions was 1.69 (2.31) per patient, 0.08 (0.12) after adjusting for the number of follow-up months. The number of readmissions in patients with >200 mL improvement in FEV_1_ was 0.04 (0.07), and in those without improvements was 0.10 (0.13) (p = 0.04).

The overall number of emergency room visits was 1.72 (2.74) per patient, 0.09 (0.14) after adjusting for the number of follow-up months. The number of visits for patients with >200 mL improvement in FEV_1_ was 0.06 (0.10), and in those without improvements was 0.10 (0.16) (p = 0.15).

Eight patients died during follow-up, one (3.7%) in the group with FEV_1_ improvement and 8 (11%) in the group without improvement (p = 0.25).

## Discussion

The present study describes changes in spirometry when measured before hospital discharge due to COPD exacerbation and in the stable phase 8 weeks after discharge. One important finding is that it is possible to perform spirometry with acceptability and reproducibility criteria during a hospital admission. Another finding isthat spirometry performed before discharge is reliable for the diagnosis of the disease with some variation in the classification of spirometric severity. However,no clinical variable indicates which spirometry parameter will improve at 8 weeks; those that do improve have a better mean term evaluation. Therefore, spirometry before discharge would be desirable to confirm the diagnosis and obtain other prognostic information at follow-up.

In previous studies, the number of patients with COPD exacerbation who were able to perform spirometry with validity and reproducibility ranged from 69% to 90% [15,17,18]. These data, converging with our own, seem to suggest that the number of adequate maneuvers increases with the number of days since admission [15]. This aspect could have influenced the high percentage of valid spirometry measures in Rea et al. [12] or our study, in which spirometry was performed around the 4th and 6th day,respectively.

In more than 90% of cases without prior confirmation, spirometry shows obstruction (data not shown), which indicates a high reliability of the clinical diagnosis in these cases, although spirometry in these cases would also be useful to estimate the degree of obstruction.

It is important to keep in mind that, in a remarkable number of patients, it is not possible to perform spirometry at 8–12 weeks of discharge (at baseline conditions) as recommended in the guidelines [1,2]due to more than a third of them presenting with new exacerbations, poor evolution of the present disease, or other problems that do not allow spirometric assessment after discharge. Cushen et al. [18] performed a functional follow-up during and after hospital admission for acute exacerbation of COPD (AECOPD); 52% of the patients presented new exacerbations. This is especially important in COPD populations at high risk of exacerbations and reduced pulmonary function, such as those included in our study and those who routinely enter the hospital [10,16,24]. The feasibility of spirometry before discharge in most patients and the difficulty performing it in the following weeks could be an argument in favor of performing this test during admission for AECOPD, asit would allow the diagnostic suspicion to be reinforced in patients without spirometric confirmation or allow the functional severity of the disease to be approximated.

Another remarkable finding in our study is that pre-discharge spirometry has acceptable reliability compared tospirometry performed at baseline, though the values are significantly lower. In the scarce studies available, the results obtained with regard to this issue are very different. Cushen et al. [18] observed an average increase of 180 cc in the first 2 weeks (from day 0 of exacerbation) in patients who do not present exacerbations after hospital discharge, but this figure seems to stabilize and not increase at day 42. However, Rea et al. [14] observed an average increase of 40 mL in FEV_1_ between hospital discharge (± 4 day of admission) and 30 days after, but this difference was not significant. In a small sample of patients, Parker et al. [16] did not observe differences in mean FEV_1_ between day 0 and 7 of AECOPD, but observed a subsequent increase of 120–130 mL on days 14 and 30, reaching 240 mL on day 60 with respect to day 0. Although all studies included patients with a mean FEV_1_ of 35–40% and ~1 L, some of these differences could be explained by the limited sample sizes of these three studies or their different designs. In addition, it could represent populations of COPD patients with different phenotypic profiles, differences in their multidimensional evaluations, or even different therapeutic management. The clinical relevance of an average increase in FEV_1_ of 80 mL after 2 months of exacerbation, as found in our study, would need to be examined. As in the study by Rea et al. [14], the increase was inversely proportional to the FEV_1_ value measured at discharge but not reached when the study population was subdivided into functional groups, though it can guide the intensity of the expected improvement.

Although there were changes in GOLD classification prior and after discharge, it was with an acceptable concordance. Given that some drugs such as inhaled corticosteroids are indicated in exacerbating patients below a level of FEV1, the variations observed in FEV1 could influence the adequate selection of the combination of inhaled drugs.

Notably, at 2 months spirometry was no longer obstructive in 2.8% of patients, which is significantly lower than in previous similar studies; Rea et al. measured a 7.4% improvement [14]. In addition, population-based studies of COPD have founda reversibility to normality of 12% at 12 years [25].

More than a quarter of patients had FEV_1_ improvements of ≥200 mL at 8 weeks. These changes have discrete but significant consequences on the clinical impact of the disease as measured by the CAT questionnaire, but not on dyspnea determined by the mMRC scale. Patients with significant improvements in FEV_1_ had mean decreases in the CAT score of approximately 3 points compared to those who did not functionally improve, which may exceed the clinically important difference described by other authors [26,27]. Although this questionnaire correlates well with recovery from exacerbations [28], our study is the first to relate it to an improvement in lung function [18]. On the other hand, we observed that an increase inFEV_1_ of ≥200 mL at 8 weeks is also related to a lower risk of rehospitalization due to AECOPD in the following months; although significant differences were found, there appears to be a small tendency for this group of patients to die less frequently. Future studies should confirm or discard this association; if these findings are confirmed, the spirometric changes at 8 weeks with respect to discharge would have relevant prognostic value, allowing patients with a progression other than the disease to be identified, which would be an opportunity for preventive intervention.

We have not found sociodemographic or clinical-functional variables that allowus to predict which patients will present an important spirometric improvement. Although without reaching a statistical significance, we observed a trend towards a relevant increase in FEV1 in active smokers and patients with a lower FEV1 prior to discharge.

We expect variables such as asthma, COPD overlap syndrome (ACOS), a previous positive bronchodilator test, eosinophils in peripheral blood, or previous total IgE to be related to a functional improvement [2] due to potentially greater functional variability. However, our data do not support this hypothesis. One possible explanation is that pre-discharge spirometry was performed almost 1 week after admission and, during that time, all patients received high doses of bronchodilators and intravenous steroids, which may have caused an improvement in function. Although, it could also be due to the fact that sample size is not enough to reach these conclusions.

Among the strengths of the present study are the high number of patients included and number of variables compared to previous studies. However, the study also has some limitations, such as being performed in a single center. Thus, our findings should be confirmed in future studies.

Our study included patients with a confirmed diagnosis of COPD before their admission and patients who did not have a confirmatory spirometric test, since the aim of this study was to assess the reliability of the spirometry performed prior to discharge, comparing it with the one performed 8 weeks after discharge; not intending to test the usefulness of spirometry during admission to confirm the diagnosis in cases with suspicion of COPD or with the diagnosis made exclusively based on clinical criteria. Although the differences were not significant, it seems that the latter present greater functional improvements, an aspect that should be taken into account and studied specifically in future studies, including only patients without previous diagnosis of COPD.

In addition, the 200 mL cut-off used in this study could be debatable for the consideration of significant spirometric improvement at 8 weeks. We performed different evaluations (data not shown) considering other cut-off points with improvements of 100 or 150 mL and 12% in FEV_1_ and FVC, but these changes do not relate to clinical changes or subsequent events as readmissions. These results confirm data from the ERS/ATS recommendations.^23^

In summary, the present study provides information about the role of pre-discharge spirometry in hospitalized patients with a diagnosis of AECOPD. Our data confirm that spirometry after several days of admission due to AECOPD is valid and reproducible in most patients, allowing us to confirm the diagnosis and contribute to its functional evaluation. The reliability of spirometry before discharge compared to spirometry performed weeks later in the stable phase is adequate, and the significant improvement in the FEV_1_ correlates with changes in the clinical impact of the disease andisa predictor of later events. If these findings were confirmed in similar studies, the data would indicate that current recommendations on the value of this test could be modified during and after the hospitalization of patients admitted for COPD exacerbation.

## Abbreviations

ACOS: Asthma-COPD overlap syndrome
AECOPD: Acute exacerbation of COPD
CAT: COPD Assessment Test
COPD: Chronic obstructive pulmonary disease
FEV_1_: Forced expiratory volume in the first second
FVC: Forced vital capacity
GOLD: Global Initiative for Chronic Obstructive Lung Disease
IC: Inhaled corticosteroid
LABA: Long acting β_2_ agonist
LAMA: Long acting muscarinic antagonist
LTOT: Long-term oxygen therapy
mMRC: Modified British Medical Research Council dyspnea scale
NIV: Non-invasive ventilation
